# Topic-Emotion Propagation Mechanism of Public Emergencies in Social Networks

**DOI:** 10.3390/s21134516

**Published:** 2021-07-01

**Authors:** Meng Cai, Han Luo, Xiao Meng, Ying Cui

**Affiliations:** 1 School of Humanities and Social Sciences, Xi’an Jiaotong University, Xi’an 710049, China; lh1129929019@163.com; 2 School of Journalism and New Media, Xi’an Jiaotong University, Xi’an 710049, China; mengxiaocntc@163.com; 3 School of Mechano-Electronic Engineering, Xidian University, Xi’an 710071, China; ycui@xidian.edu.cn

**Keywords:** public emergency, information propagation, social network analysis, topic recognition, emotion analysis

## Abstract

The information propagation of emergencies in social networks is often accompanied by the dissemination of the topic and emotion. As a virtual sensor of public emergencies, social networks have been widely used in data mining, knowledge discovery, and machine learning. From the perspective of network, this study aims to explore the topic and emotion propagation mechanism, as well as the interaction and communication relations of the public in social networks under four types of emergencies, including public health events, accidents and disasters, social security events, and natural disasters. Event topics were identified by Word2vec and K-means clustering. The biLSTM model was used to identify emotion in posts. The propagation maps of topic and emotion were presented visually on the network, and the synergistic relationship between topic and emotion propagation as well as the communication characteristics of multiple subjects were analyzed. The results show that there were similarities and differences in the propagation mechanism of topic and emotion in different types of emergencies. There was a positive correlation between topic and emotion of different types of users in social networks in emergencies. Users with a high level of topic influence were often accompanied by a high level of emotion appeal.

## 1. Introduction

With the development of the Internet, social networks based on social media have become the main channel for the public to share their daily life, express their views, and interact with each other. The breaking of the time and space restrictions of social media leads to the rapid spread of public emergencies, making social networks become the “virtual sensor” of various public emergencies [1]. When a public emergency occurs, the public tends to share their attitudes, views, and comments on social networks. Through data mining in social networks, we can obtain valuable public emergency information, such as the emotion, topic, and social change, which is advantageous to the multi-dimensional crowdsensing of events, as well as the emergency management of auxiliary events to maintain the stability of social order.

On the other hand, with the development of the economy and the transformation of society, social problems and contradictions around the world have become increasingly acute, which lead to all kinds of public emergencies from time to time. When a public emergency is formed, to reduce uncertainty, people tend to have a strong demand for information on the development of the event and policy response and try to obtain relevant information from social networks [2]. However, due to the sudden, immediate, and uncertain characteristics of public emergencies, the information transmission process of the events also presents the characteristics of the random disorder, agglomeration, and looseness. At the same time, due to the lag of the government response to the event and the inappropriate media reports, the information held by all subjects in the event is unequal and asymmetrical. The panic and anxiety emotions, as well as rumors, tend to spread, making the online expression more emotional and extreme [3].

Since the birth of the concept of Web2.0 in 2004, social media has not only opened up a new space for information exchange and opinion expression but also served as an important driving factor for social reform [4]. According to the latest statistics released by We Are Social and Hootsuite, there have been 4.2 billion Social media users worldwide in 2021, accounting for more than 53% of the world’s total population [5], which means social media has been widely used worldwide. Through the use of Twitter, Facebook, Weibo, and other social media software, people can conveniently share their experiences and ideas on the social media platform, and forward others′ views and contents [6]. It is the universality, interactivity, and real-time characteristics of social media that make social media become an important communication mechanism for the public to quickly and widely disseminate information, express opinions and emotion when facing public emergencies [7]. When a public event occurs, the public quickly establishes a cooperative communication network through social media [8]. Through the realization of such functions as posting, commenting, and forwarding, a complex topic, and emotion communication network is constructed in a short time [9]. At the same time, social media is not only an important platform for the public to understand and exchange information related to the event and seek official explanation, but also an important means for the government to respond to social demands and calm the public emotion. Therefore, social media naturally becomes the focus of research [10].

The development of social media technology has broken the limitation of media, space, and time, and the social network built through social media has deeply influenced society. However, the lack of applicable laws and regulations in social media platforms and the imperfection of management and organization systems make the current cyberspace full of disorder, distortion, and other negative characteristics. People use various media to spread the information they like or dislike, which makes people enjoy the pluralistic and open atmosphere of the information society, but also face the objective risks brought by the network public opinion. When public emergencies occur, the public tends to lack thinking about the facts in a short time and be carried away by irrational negative remarks, resulting in emotional excitement, a sharp increase in pressure, and magnified anxiety. At this time, the public tends to seek resonance in social networks, but under the effect of group polarization, it is easy to be bound by the majority of groups and go to extremes, endangering public safety and social stability. The characteristics of social media, such as unstructured, spontaneous, large user base, fast transmission speed, many irrational emotions, and group polarization, also increase the difficulty for the government to manage [11]. Therefore, it is of great practical significance to study the topic and emotion propagation of public emergencies in social networks. In view of this, two research questions are addressed:

(1) What are the commonalities and differences of different types of emergencies in the network propagation process of the topic and emotion?
(2) What is the relationship between the topic and emotional propagation of different types of public emergencies in social networks?

To solve the above questions, this study investigates the generation and propagation characteristics of topic and emotion content of multiple subjects in different public emergencies based on social network analysis, knowledge discovery, and deep learning approach, and constructs the topic and emotion propagation maps of different types of public emergencies in social networks.

The rest of this paper is structured as follows. In Section 2, we will review relevant studies on public emergencies, emotion analysis, and social network analysis. Section 3 will create a “Preliminaries” section to explain the techniques that we used in paper and explain how and why we decided to use those techniques. Section 4 will introduce the research design of this paper, including text data preprocessing, topic recognition, and emotion analysis methods. Section 5 will introduce the research implementation of this paper, construct the topic and emotion evolution maps of different types of public emergencies, and describes the evolution characteristics of the topic and emotion. Section 6 will conclude the results and the future research direction is put forward.

## 2. Related Work

### 2.1. Applications of Deep Learning Method in Social Network Analysis

In order to process large amounts of information to achieve the expected research results, machine learning algorithms (or pattern recognition and data mining [12]) are usually used to provide high-precision results, such as decision trees, support vectors, or artificial neural networks [13]. However, with the advent of the big data age, the traditional machine learning method has limited ability in processing raw data. The deep learning method emerges as the times require [14], which is widely used in speech recognition [15], image recognition [16], and text classification [17].

Deep learning has outstanding performance in various research fields, including related research based on social networks [18]. With Facebook, Twitter, and other social platforms becoming an integral part of people’s daily life, their information has become efficient data sources for researchers to study social networks using deep learning methods [18,19,20]. Through the analysis of the social network data, researchers can summarize the interests and views of each user (node), find the relationship characteristics from the communication (link) between users (nodes), and study the communication of public events in social media platforms. For example, the graph convolutional network (GCN) was used to investigate how user preferences are influenced by social communication. Online advertising targeting [21], personalized recommendation [22], infectious marketing [23], social health assistance [24], social influence analysis [25], and academic network analysis [26] were also in concern.

Deep learning also has outstanding performances in natural language processing (NLP), especially topic classification, emotion analysis, question answering, and language translation. For example, based on CNN, the malware system call sequences were modeled to classify malware [27]. The word embedding was trained with the unsupervised neural language model to analyze the Twitter text data on a large scale [28]. Some researchers modeled low-dimensional embeddings of words and knowledge base constituents to score the natural language processing questions of the answers [29]. The neural network machine translation method could further improve the translation performance [30]. The emotional analysis method is in line with the development of social media such as Twitter, Facebook, and Weibo, which has become the main method in social media research [31].

Emotion refers to people′s subjective feelings towards objective behaviors, while sentiment is the attitude, thought, or judgment caused by feeling. The sentiment is a further reflection of emotional polarization [32]. At the same time, the method of sentiment analysis is often used in the analysis of emotion, so we have also carried on the related elaboration to the sentiment analysis. Sentiment analysis is a computational study on the opinions, emotions, evaluation, and attitudes of products, services, organizations, individuals, events, topics, and other entities, involving emotional classification, opinion information extraction, opinion summary, emotional retrieval, and other NLP tasks [33]. As an important part of NLP, sentiment analysis is widely used in data mining, web mining, text mining, and information retrieval. For example, comparing the performance of different machine learning algorithms on user age predicting with the help of analysis method [17,20]. Some researchers combined the deep learning text classification and social network analysis method to analyze the content and communication structure during the public emergency [34].

### 2.2. The Public Emergency Propagation in Social Networks

Compared with traditional media, social media has an advantage in collecting and delivering messages. By using social media such as Twitter and Weibo, the public can publish, share and disseminate information in real-time [35,36]. This also endows social media with the ability to build a collaborative contact network in the event of unexpected public events and provides a way for the public to timely receive the official and unofficial information related to public events [37]. It is also the advantage of social media in information communication that makes it play an increasingly important role in the communication process of public emergencies, which attracted the attention of many researchers [38,39]. At present, the focus of public emergencies on social media is mainly on information dissemination content, key node recognition, stakeholder response, and other aspects [40,41,42]. For example, a previous study used the content analysis method to confirm that people increasingly relied on social media to obtain relevant information about public emergencies, and displayed emotions by publishing specific information content [43]. Large-scale text analysis of social media data was used to identify the needs and responses of specific groups to achieve effective emergency responses [44].

In recent years, social networks based on social media platforms have been widely used, among which the monitoring of unexpected public emergencies is one of the most common applications [45]. In this process, social networks are often regarded as sensors of public events because of their big data availability, information processing, forwarding, and other processing functions [46]. Related studies analyzed four types of public emergencies: natural disasters, accident disasters, public health events, and social security events. For example, text features in social networks were analyzed by the probabilistic spatiotemporal model to infer the center and influence range of earthquakes [47,48]. Disaster information in social networks was analyzed to help life-saving and rescue activities [49,50]. Social networks can not only be used as data sources for public health monitoring but also be used as a communication tool to spread disease risk and intervention measures, promote healthy lifestyles and publicize health policies [51,52]. In social security incidents, social networks were used to get emotional perceptions with the methods such as data mining and information retrieval to assist governments, enterprises, and other organizations [53,54]. In short, social networks have changed the tradition that public emergencies can only be transmitted in one direction [55], which provides a new situation for the public emergency formation and disposal.

### 2.3. The Public Emergency Emotion in Social Networks

The frequency of public emergencies is increasing during the social transformation. The controversy of public emergencies is often accompanied by a variety of emotions [56]. Therefore, the emotion analysis of public emergencies in social networks is of great significance to understand public views, improve emergency management, and innovate social governance [57]. The related research mainly focused on the influencing factors and the evolution and diffusion law of public emotion in public emergencies. In the aspect of influencing factors of public emotion, previous studies mostly explored the content and form of information release, communication subject, and channel. For example, relevant studies have found that when government information release was slow, opaque, poor coordination, and other characteristics, negative emotions would spread rapidly [56]. At the same time, there are also studies on the sentiment of the content released by the public [58]. Through the use of sentiment analysis and data mining methods, we can know how the authenticity of information affects sentiments. The results show that when the information was misleading, the audience tended to have negative sentiment. The misleading information with negative sentiment has more transmission than that with positive sentiment [59]. In addition, the emotion expression of groups with different characteristics in social networks is also different. Anonymity significantly affected the release of information and emotional expression of the public in social networks [60,61]. Anonymous users showed a significantly higher expression frequency of extreme and negative emotion than non-anonymous users [62].

On the other hand, the emotion under the public emergency evolution law of diffusion in social networks was well concerned. For example, some studies adopted the methods of time analysis and inferential statistical analysis and found that the sentiment evolution in social networks was consistent with the development of the events. Under different event topics and lifecycle, the sentiments presented different changes [63]. A previous study on social security found that when the information was not disclosed, public emotion was easy to be affected by the most significant opinions in the field of public opinion. Emotion subsided as attention diminished over time [64]. From the perspective of emotion diffusion, some studies found that information content with intense emotion in the public emergency was more likely to become the focus of people′s attention than neutral information content, and can be retweeted more quickly and frequently [65]. Some studies paid attention to the strategies that governments used after public emergency emerging and found that slow and opaque information release would promote the spread of negative emotions in social networks [66]. These studies provide useful insights for easing public emotion as well as emergency management.

To sum up, the propagation mechanism of public emergencies in social networks has become a hot topic of academic attention. Some explorations have been made on the influencing factors of the propagation process, which provides a basis for this study to explore the topic and emotion propagation of public emergencies in social networks based on the deep learning method. At present, research on emergencies in social networks mostly emphasize the influence of government departments as the main body of information release, while the spreading mechanism of topic and emotion under different user types in social networks has not been highlighted. Existing studies mostly choose a single dimension of emergencies, with little consideration given to the differences in topic and emotion propagation of different types of emergencies in social networks. These are the directions to be explored in this study.

## 3. Preliminary

### 3.1. Topic Recognition with the Deep Learning Method

The method of extracting valuable topics that can arouse public interest from big data is called topic recognition. Topic recognition is widely used in public opinion analysis, news lead tracking, business analysis, and other fields [67]. Existing studies have mainly adopted two methods for topic recognition text information. On the one hand, cluster analysis is conducted based on the detection of characteristic words or phrases related to public events. On the other hand, a topic model capable of recognition is established by revealing potential topics in a large number of documents [67]. However, social media is full of useless information, which makes it difficult to cluster emerging features only by cluster analysis method [67], and it is not ideal to directly apply the traditional topic model to the information content of short texts [68]. With the development of deep learning methods, Word2Vec and other text topic mining methods have achieved good results in topic recognition of text data in social media [69].

**Figure 1:**
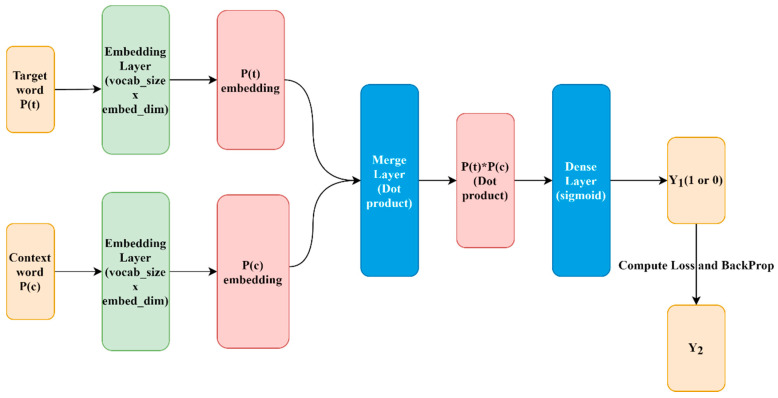
The structure of Skip-gram model.

Word2vec is a word embedding method proposed by Google in 2013, which is a shallow and two-layer neural network language model. Based on a given corpus, words can be transformed into word vectors more effectively through the optimized training model [70]. Through training in a large-scale text dataset, the model maps text content into a k-dimensional vector space and further qualitatively measures the similarity between words in the word vector space [71]. Among them, Word2vec mainly includes two learning algorithms, namely, Continuous bag-of-words and Continuous skip-gram. The former uses a continuously distributed representation of the context, predicting the current value from context words, and is most commonly used in small datasets. The latter is different from the former in that it predicts contextual words based on the current word and is often used in the analysis of large-scale text datasets [71,72]. In this study, the Skip-gram model is used for the topic analysis of text data, and the model structure is shown in Figure 1. The input of the model is the target word and the context word or the random word pair, which is passed to the embedding layer initialized with random weight to obtain the word embedding of the target word and the context word. Then, the word embedding representation of the two is passed to a merged layer for dot product operation, and the dot product value is passed to a dense layer using the sigmoid activation function. The layer outputs the predicted value (Y1) of whether the pair of words are contextual or random. Then, the outputs are compared with the actual label (Y), and the loss is calculated through the mean square error. The embedding layer weight is updated by backpropagation.

The Skip-gram model is mainly used to predict the context of the current word or sentence. $P\left(t_{i}|t_{n}\right)$ is the prediction probability. $t_{n}$ represents the current word. u refers to the context parameters (n−u≤i≤n+u). The greater the u, the more the number of contextual words, the higher the accuracy of the training, the training time is longer. With a given training word sequence $t_{1},\;t_{2},\;t_{3},\;\cdots \cdots t_{N}$, the maximum objective function of the Skip-gram model is:

(1) $$\frac{1}{N}\sum_{n=1}^{N}\sum_{u\leq i\leq u,i\neq 0}\log P\left(t_{n+i}|t_{n}\right)$$

The basic Skip-gram model is defined as $P\left(t_{s}|t_{m}\right)$. $\omega_{t}$ and $\varphi_{t}$ are input and output variables, respectively. The size of the dictionary is T. The predictive probability of $t_{s}$ generated by the keyword $t_{m}$ is as follows:

(2) $$P\left(t_{s}|t_{m}\right)=\frac{\exp\left(\varphi_{t_{s}}^{N}\;\omega_{t_{m}}\right)}{\sum_{t=1}^{T}exp\left(\varphi_{t}^{N}\;\omega_{t_{m}}\right)}$$

### 3.2. Emotion Analysis with Deep Learning Method

Traditional content analysis methods are limited by human cost and other factors when faced with large-scale social media data, so computer-aided emotion analysis methods are widely used. Commonly used emotion analysis methods mainly include emotion computation based on emotion dictionary and classification algorithm based on machine learning technology [73,74]. The lexicon-based approach mainly determines the emotion orientation of the opinion words through the emotion labeling, while the machine learning method uses a part of the complete data to train the classifier, independent of the previous vocabulary. Typical methods include Naive Bayes, maximum entropy, and support vector machines. In recent years, more and more scholars have adopted the deep learning model as a classifier and achieved good results [75,76].

In related studies on emotion analysis using deep learning models, recurrent neural networks (RNNs) that are capable of processing sequence data such as text are the most common. In order to overcome problems such as gradient disappearance, gradient explosion, and short-term memory, researchers proposed the long short-term memory (LSTM) model [77]. LSTM mainly consists of three gates (input gate, forgetting gate, and output gate) and a memory unit. “Gate” is a way to let information selectively through, in which the forgetting gate determines what information should be forgotten by the memory unit, the input gate is the input to the current information, determines the information stored in the memory unit, and finally the output gate determines the output content of the memory unit to the next state. Through the use of input gate, forgetting gate, and output gate, LSTM can delete or increase the information in the state of the memory unit, and effectively solves the problems of gradient vanishing, gradient explosion, and long-term dependence existing in RNN. The specific calculation formulas of LSTM are as follows:

(3) $$f_{t}=\sigma \left(W_{f}Z_{t}+W_{f}h_{t-1}+b_{f}\right)$$

(4) $$i_{t}=\sigma \left(W_{i}Z_{t}+W_{i}h_{t-1}+b_{i}\right)$$

(5) $$c_{t}=f_{t}c_{t-1}+i_{t}tanh\left(W_{c}Z_{t}+W_{c}h_{t-1}+b_{c}\right)$$

(6) $$o_{t}=\sigma \left(W_{o}Z_{t}+W_{o}h_{t-1}+b_{o}\right)$$

(7) $$h_{t}=o_{t}tanh\left(c_{t}\right)$$

where $h_{t}$ represents the hidden state of the memory unit at time t. σ is the sigmoid function. $Z_{t}$ is the input of word vector at time t. W and b represent weight and deviation, respectively, to be trained in the LSTM model.

**Figure 2:**
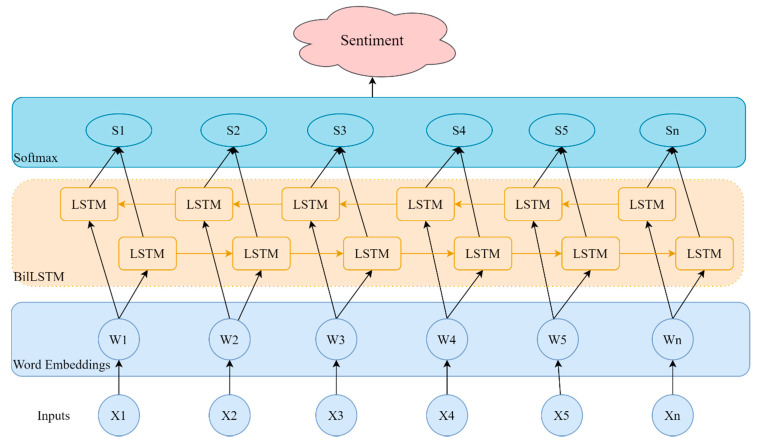
A biLSTM network for emotion classification.

The emergence of LSTM solves the problems faced by traditional RNN, but LSTM adopts a front-to-back order in the processing of sequence data, ignoring the future characteristics of sequence data (through the backward state). Therefore, the bidirectional LSTM (biLSTM) model is proposed. Compared with the LSTM model, the biLSTM model combines the information in both forward and backward directions of the input sequence, so it has more advantages in short text emotion analysis [78]. The structure of the biLSTM emotion classification model is shown in Figure 2. Firstly, the words contained in the text are input into the pre-trained word embedding layer to obtain the word vector representation. Then, word vectors are input into the biLSTM layer in order to obtain the hidden forward and backward states. Finally, the hidden states in all directions are spliced, input to the full connection layer containing the softmax activation function, and output the predicted probability distribution on the emotion categories.

In addition, the pre-training model can make use of massive unlabeled corpus data, which are widely used for a variety of natural language processing tasks. By fine-tuning the pre-training model, a better result of emotion classification can be achieved. Pre-training is generally divided into two steps. First, the model is trained with a large dataset; the next step is to transform the pre-training model according to different tasks and fine-tune the pre-training model with the dataset of this task.

## 4. Methodology

**Figure 3:**
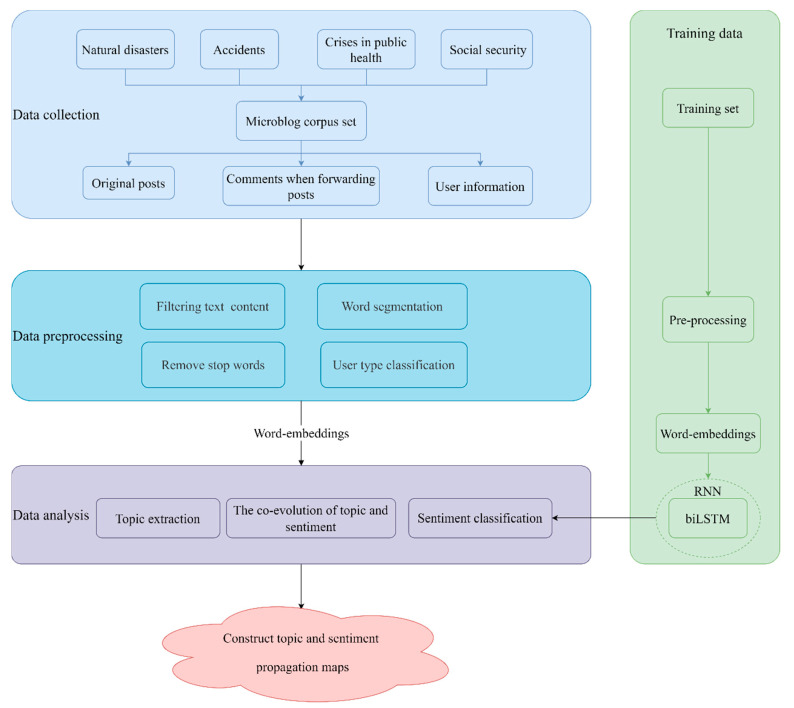
Research framework.

In this study, natural disasters, accidents and disasters, public health, and social security incidents were selected as examples. Python software was used to collect the microblog corpus published by users. After that, text content was filtered, stop words were removed and user types were classified. In terms of data analysis, we analyzed the propagation mechanism of topic and emotion in social networks when public emergencies occurred. The topics were identified through the Word2Vec and K-means clustering method. The emotion of posts was classified by the bidirectional long and short-term memory mode. Finally, we explored the law of the co-evolution of topic and emotion in social networks through the concepts of topic out-degree and emotion out-degree, and used the topic and emotion propagation maps to visualize. The research framework is shown in Figure 3.

### 4.1. Data Collection and Preprocessing

This paper took public emergencies as the research object. According to the types of emergencies, public emergencies can be divided into four types: natural disasters, accidents and disasters, public health events, and social security events. In order to compare the propagation mechanism of different types of emergencies in social networks. This study selected one case of each type that happened in recent years, including the fire in Liangshan, Sichuan Province (natural disaster), the collapse of the hotel isolated from the epidemic in Quanzhou (accident disaster), the epidemic of the Diamond Princess cruise ship (public health) and the hostage-taking of students in Kunming (social security). Sina Weibo platform was selected as the data source to gather emergencies related text data.

Sina Weibo is one of the most popular social media platforms in China. According to Weibo′s financial report for the fourth quarter of 2020, the Weibo platform had 221 million monthly active users and 225 million daily active users in December 2020, making it one of the most widely used social media platforms in China. Through the use of Weibo, users can thumb up, comment, and forward any news or event, and build a huge social network through interaction in the social media platform. The four types of public emergencies selected have all become hot topics in Weibo, attracting the attention of a large number of users. Through the interaction and discussion among users, a large number of event topics, emotion data have been generated. Based on this, we gathered the emergency-related information by limiting the period and searching keywords. Each post included the content, the thumb up, comment, forwarding, as well as the publisher′s personal information.

After the event-related text data were obtained, the data need to be preprocessed. The pre-processing methods adopted in this study are as follows. Firstly, regular expressions were used to filter non-text contents in text data, such as punctuation marks, HTML tags, etc. Secondly, since there was no space between Chinese statements, the study uses Jieba word segmentation to carry out word segmentation processing, so as to convert them into word vectors later. Finally, the Chinese stop word list provided by HIT was used to remove meaningless words. In addition, Sina Weibo users have multiple authentication types that indicate the user′s status, role, or affiliation. Considering the difference in status and influence of different types of users, this paper divided the user types into five categories according to the authentication type field of user information: ordinary users, celebrities, government, institutions, enterprises, and media.

### 4.2. Clustering of Microblog Topics in Emergencies

In this study, we used Word2vec and K-means to cluster the topic of various types of public emergencies in social networks. Firstly, in order to reduce the interference of useless information and noise in the Weibo text data, the TF-IDF method was used to calculate the importance of each word in each microblog file for the document representation. The top 20 words in TF-IDF weight are extracted from the largest to the smallest as the keyword representation of the microblog file. Then, the Skip-gram model was used to convert the unstructured microblog text into a computable and structured word vector. With the help of Gensim toolkit of Python language, the tokenized words are transformed into a 100-dimensional word vector. After that, for each public emergency, the K-means clustering method was used to cluster word vectors into topics. Each topic was represented by a series of keywords, which were further summarized into topic descriptions. Considering the influence of K value selection on the results, this study selected appropriate K values for different emergencies according to work requirements and repeated practice modifications.

### 4.3. Fine-Grained Emotion Classification of Microblogs in Emergencies

NLP&CC2013 and NLP&CC2014 datasets were used for Weibo sentiment classification task for the International Conference on Natural Language Processing and Chinese Computing, which has been widely used in the training and evaluation of Chinese sentiment classification models in recent years [79,80]. Because the emergency data of this study were also from the Weibo platform, and in order to fairly evaluate the performance of the sentiment classification model used in this paper, the NLP&CC dataset was chosen as the training and test data. Each post in the datasets has emotion labels. There are 7 emotion labels, except for None, which are ”Like”, ”Disgust”, ”Surprise”, ”Happiness”, ”Fear”, ”Sadness”, and ”Anger”, separately. Microblogs containing the above 7 emotion labels were extracted and combined into the dataset. The dataset contained a total of 22,641 microblogs, 80% of which were used as the training set to train the model, and the remaining 20% as the test set to evaluate the prediction performance of the model.

In order to improve the performance, as well as the generalization ability on unfamiliar datasets of the model, the pre-train model of microblog was established before the emotion classification model was trained [81]. Therefore, 200,000 microblogs filtered were randomly gathered, and the skip-gram method of Word2vec was used for pre-training. This makes the sentiment classification model not only consider the text in the annotated dataset, but also the text in the larger unannotated microblogs. Then, the emotion classifier composed of the biLSTM model was connected to embedding layer to train and predict the emotion on the NLP&CC dataset. Finally, the emotion classification accuracy of this seven-classification model on the test set was 0.71, which met the needs of this study for emotion classification.

### 4.4. Visualization of the Network Propagation of Topic and Emotion in Social Media

Based on the obtained data, Gephi software was used to construct the propagation maps of topic and emotion in social networks. In the construction of the topic and emotion propagation maps, the study defined a single Weibo user who forwards relevant information on the Weibo platform when an unexpected public event occurred as a “node”. The forwarding relationship between any two Weibo users in social networks in the transmission process was set as an “edge”. The structural attributes of forwarding networks related to different types of public emergencies were presented through network density, vector centrality, clustering coefficient, and other indicators.

In order to further explore the evolution mechanism of topic and emotion in social networks, researchers defined the influence of topic and emotion in social networks as topic out-degree and emotion out-degree, respectively, based on existing studies. Among them, the topic out-degree was explained as the proportion of the same type of topic among all the nodes extending outward from the node in social networks built on the forwarding relationships. The emotion out-degree was interpreted as the proportion of the same type of emotion among all the nodes extending outward from the node in the social network formed by the construction of forwarding relationships [9]. The former mainly represents the influence caused by the spread of topics in social networks, while the latter mainly represents the influence caused by the spread of emotion in social networks. Based on the calculation of topic and emotion out-degree, the propagation map of different nodes in the social network constructed by forwarding relationship was drawn in the public emergency. Through text data mining in social media, this study constructed the topic and the emotion propagation map of the social network under public emergencies. Based on the network topology analysis of the different types of public emergencies in social media, the propagation characteristics of public emergencies in social networks were further revealed.

## 5. Findings and Discussions

### 5.1. Descriptive Analysis

The four types of public emergencies selected in this paper had all caused great repercussions in cyberspace and attracted the attention of the public. Internet users provided us with a wealth of topic and emotion data by posting, commenting, and forwarding on social media platforms. In this study, the forest fire in Liangshan, Sichuan Province, which happened on 30 March 2019, was selected as a natural disaster case. The accident occurred in Muli County, Liangshan Prefecture, Sichuan Province. The sudden forest fire caused a total of 31 deaths, of which 27 were local firefighters; The collapse of the quarantine hotel in Quanzhou City, Fujian Province on 7 March 2020, was selected as the accident disaster case. A total of 71 people were trapped at the time of the collapse, most of which were people who had been under quarantine observation in the hotel during the epidemic period. After the rescue, 42 people were finally rescued; as public health care, we selected the outbreak on the Diamond Princess ship on 1 February 2020. Some of the passengers on the Diamond Princess ship with more than 3000 people on board were detected to have COVID-19 and spread on the ship; the case of hostage-taking in Kunming, Yunnan Province on 22 January 2021, was selected as a social security case. A man with a knife wounded seven people at the gate of a local middle school and then took a student hostage. These incidents had been widely discussed on social media since their occurrence. By searching event hashtags, we found relevant discussions of events in social media, and collected the interactions of different types of emergencies in social networks under the condition of limiting the active time of the events, to obtain the microblog entries and forwarding numbers related to the events. The number of microblog entries and the Forwarding relationship of each event is shown in Table 1.

In the visualization of distribution and propagation, it was concerning that too many users participated in discussion and interaction when public emergencies occurred, and different types of public emergency datasets had differences in text quantity and spatial distribution. We selected different criteria to screen the number of nodes that constitute the spectrum for different types of public emergencies. For example, in the case of the natural disaster, the study selected users whose microblog entries were forwarded reached four times or more as nodes and then constructed the propagation maps of related topics and emotions in social networks when the natural disaster occurred. The specific filtering criteria is shown in Table 2. Among them, the quantitative information of various types of Weibo users in the propagation process of public emergencies is shown in Table 3.

It can be seen from Table 3 that celebrities and media occupied a large number of nodes in various types of public emergencies and occupied a favorable position in social networks. They were important channels for the dissemination and diffusion of topic and emotion in social networks. The government has a large number of nodes in the natural disaster and accident, but a small number in public health and social security event. It means that when different types of emergencies occurred, the role of the government in social networks was different. Ordinary users played a relatively stable role in social networks composed of different types of emergencies. A small number of influential ordinary users together with celebrities, media, and other authenticated users jointly form the transit channel for the dissemination of topic and emotion information in social networks and occupied an important position. Most ordinary users become the receivers of topic and emotion information due to their lack of influence. The number of nodes of institutions and enterprises in various types of emergencies was small and does not reflect obvious propagation characteristics.

Table 4 shows the indicators of social networks formed by forwarding relationships when different types of public emergencies occurred, reflecting the differences in structural attributes of forwarding networks formed by different types of emergencies. The average degree was calculated by dividing the number of edges by the number of nodes. The results show that the average degree of the social security event was the largest and the graph was denser, while the average degree of the natural disaster event was the smallest and the graph was the sparsest. The network diameter was the distance between the two nodes with the longest shortest path in a network. The results show that the network diameter of the social security event was the largest, with a value of 8. The network diameter of the other three events was all 3. Modularity is a method of cluster analysis. The quality of clustering was represented by modularity and distributed in the range of 0–1. The larger the number, the better the clustering effect. The results show that the four types of emergencies were all modularized to a certain extent, and the network tended to be small groups rather than random. The clustering effect of the public health event was the best. According to the connection relationship, the graph was divided into several blocks, and each block was called a connected component. In the directed graph, the graph was divided into a strongly connected graph and a weakly connected graph. The results show that in all types of public emergencies, strong connectivity occupied the main position. The average eigenvector centrality was calculated by the centrality of neighboring nodes. The results show that the nodes in the social security event have the highest centrality of the average eigenvector. The average path length was obtained by dividing the number of nodes by the sum of the shortest paths of all two nodes. The results show that the average path length of the social security event was the largest, and each average shortest path can be allocated to about 2.153 nodes. In conclusion, compared with other types of public emergencies, the social security incident was in a dominant position in network attributes such as average degree, modularity, and eigenvector centrality, and the public was more closely connected to social networks formed by the social security incident.

### 5.2. Topic Recognition and Propagation Visualization

Table 5 shows the topic clustering results of various types of public emergencies. For example, the topics of the natural disaster event were summarized into five categories based on the corresponding text content to obtain a better topic clustering effect, and the topics and some high-frequency feature words were listed, respectively. The feature words of the first topic in natural disaster event were “Kindling”, “compatriots”, “consequences”, “platform”, “diary”, “loss”, “doubt”, “Wanting”, “Heroic”, etc., which was summarized as “Condemning the net users who insult firefighters”. The topics of the natural disaster were mainly about the mourning for the sacrificed firefighters and the concern for the physical and mental health of the surviving firefighters. The accident event mainly focused on the investigation of the accident and the subsequent search and rescue situation. In addition to the relationship between those infected by the epidemic, the topic of the public health event had focused on the blame for the lack of responsibility of those responsible. The topics of the social security event mainly focused on the praise of the hero of rescuing the hostages, the condemnation of the prisoners, and the concern for the victims and their families.

**Figure 4:**
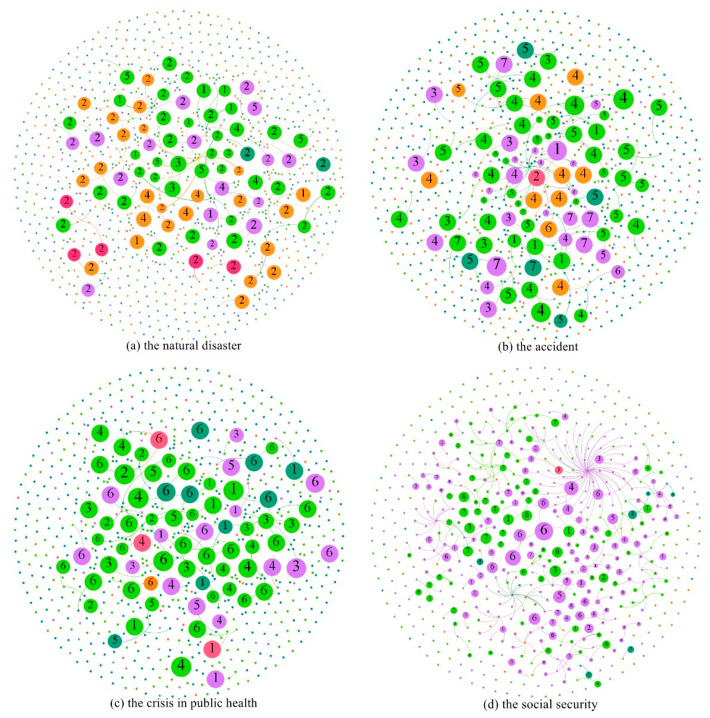
Topic propagation maps of four events. In the figure, ordinary users are represented in purple, celebrities in light green, the government in orange, institutions and enterprises in light red, and media in dark green.

Based on the obtained text data, we used Gephi software to build the propagation maps of event-related topics in social networks when different types of public emergencies occurred. In the construction process of the graph, nodes, and edges needed to be arranged by algorithms to meet the needs of the rationality of graph requirements and visual recognition. Different types of algorithms correspond to different layout methods in Gephi. According to the content needs, we chose to use the Fruchterman Reingold algorithm to layout and set the layout attributes, and finally constructed the propagation maps of the topics. The propagation maps of four different types of public emergency event-related topics in social networks were shown in Figure 4, where node label represents a topic type, node color represents user type, node size was defined as PageRank index representing node importance. From Table 3, we can see that when different emergencies occurred, there were differences in the influence and scope of influence of the event, which was externally manifested in the difference in the number of nodes. As shown in Figure 4a, the topic map of the natural disaster event contained a total of 975 nodes and 92 edges. The nodes involved influential ordinary users, celebrities, media, and governments, etc., forming a communication system with rich levels in social networks and promoting the dissemination of the topic in social networks. As shown in Figure 4d, the social security event contained 741 nodes and 425 edges. Compared with Figure 4a, Figure 4d has a smaller number of nodes, but more connections and closer connections between nodes. On the one hand, this was because the difference in the spatial distribution of the social security event was more extensive, and the screening criteria of nodes are higher, leading to a small number of nodes. On the other hand, compared with the natural disaster, the social security event was mainly composed of celebrities, ordinary users, and a small number of media users in the forwarding network. Among the certified users, opinion leaders such as governments and institutions paid less attention, which externally affected the level of event transmission and made users more closely connected.

Through the observation of the diffusion process of related topics in social networks after the occurrence of different types of public emergencies, we found that the most prominent topic category in the natural disaster event was the memory and tribute to fire heroes, followed by the related topic of condemning the netizens who insulted firefighters, which also attracted the public′s attention on social networks. When the accident happened, the shock of the collapse and the search and rescue at the Quanzhou Hotel became a major topic of public attention on social media. When the public health event occurred, the type of topic that gets the most public attention on social networks was the confirmation of COVID-19 infection among ship travelers. When the social security event occurred, the public′s interaction in social networks mainly revolved around the condemnation of the prisoners, sympathy for the abducted children, and the police kneeling. Secondly, the denial of the rumors by the families of the hostages had also aroused wide discussion among users in social networks. This also confirms that when public emergencies occurred, users in social networks paid the most attention to the specific information related to the event and the relevant situation involving the main characters. The relevant findings have guiding significance for public emergency management in case of emergency.

### 5.3. Emotion Analysis and Propagation Visualization

**Figure 5:**
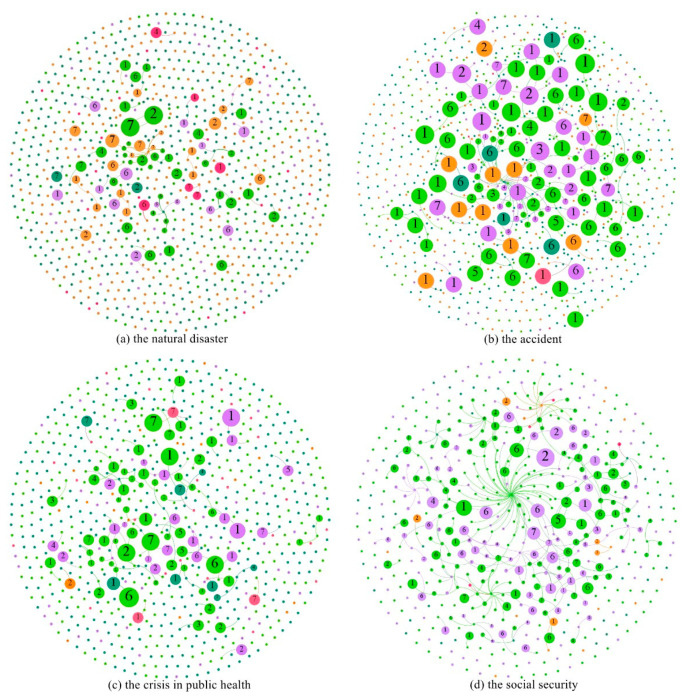
Emotion propagation maps of four events. Label 1 represents the emotion of “Like”, Label 2 represents the emotion of “Disgust”, Label 3 represents the emotion of “Surprise”, Label 4 represents the emotion of “Happiness”, Label 5 represents the emotion of “Fear”, Label 6 represents the emotion of “Sadness”, and Label 7 represents the emotion of “Anger”.

In this study, the post text of different types of public emergencies in social media was collected, and the experimental text data were preprocessed based on emotion labeling of the training set data. After the cleaning, the text data were converted into word vectors by the Skip-gram model, and the emotions contained in the input vectors were classified by Bidirectional Long and Short-Term Memory model. The emotions can be divided into seven categories, which are “Like”, “Disgust”, “Surprise”, “Happiness”, “Fear”, “Sadness”, and “Anger”. Similarly, Gephi software was used to generate the emotion propagation maps in social networks when different types of public emergencies occurred, and the Fruchterman Reingold algorithm was used for layout. The emotion maps are shown in Figure 5. The size of the node represents the PageRank index of the node, namely the importance of the node. The color and label of the node represent the user type and emotion type in social networks, respectively, when a public emergency occurred. The emotion evolution in social networks is different in different types of public emergencies. As shown in Figure 5a, the figure shows that when a public emergency of natural disasters occurred, the negative emotion of the public was high. In the figure, the public′s emotions were mainly reflected in negative emotion, such as disgust represented by Tag 2, sadness represented by Tag 6, anger represented by Tag 7, etc. Combined with the content of the text, the disgust mainly came from the resistance to the casualties and property damage caused by forest fires. When the accident occurred, the emotions of like and sadness played important roles in social networks. Combined with text content, the emotion of like came from network users’ recognition of processing mode and processing speed. Sadness came mainly from sympathy for the trapped and the dead. When the public health event occurred, as shown in Figure 5c, emotions are mainly manifested by liking, sadness, and anger. The emotion of like mainly came from the recognition and support for the control of the epidemic in their own country. Sadness came mainly from the infection of ship tourists, and anger was largely due to the ship’s managers’ failure to prevent the COVID-19 outbreak quickly enough. When a social security event occurred, emotions such as disgust and sadness became the mainstream emotions of the public in social networks. Among them, the emotion of like mainly came from the admiration for the hero and the joy and satisfaction of the hostage being rescued. The emotion of disgust came mainly from armed assault, hostage-taking, and sadness stems mainly from the harm done to the victim. What they have in common is that no matter what type of public emergencies, sadness, anger, and other negative emotions always occupy a major position in the spread process of social networks. Negative emotion spread more prominently in social networks.

On the other hand, by mapping the evolution of emotions in social networks, we also found differences in user types when different types of public emergencies occurred. As can be seen from Figure 5, the types of users in the emotion evolution of the social security event are significantly less than those in other types of emergencies. On the one hand, this was because the forwarding network in the social security event was too centralized, and the user types of the key nodes were more homogeneous. On the other hand, in the process of emotion propagation, compared with the social security event, other types of public emergencies involved a wider range of people and had a greater influence on society, thus forming a more diversified communication mechanism.

### 5.4. Topic Influence and Emotion Contagion

In the research design, we defined the topic out-degree and emotion out-degree of nodes as the influence of topic and emotion in social networks. Furthermore, the diffusion mechanism of the topic and emotion of public emergencies in social networks was further analyzed. Considering that there were a large number of nodes in social networks when public emergencies occurred, we selected the top 30 core nodes in the ranking of node out-degree for display. The horizontal and vertical coordinates represented the topic out-degree and emotion out-degree, respectively. The node color represented the user type of the node. Through the analysis of the topic out-degree and emotion out-degree of the core nodes, we could not only distinguish the differences in the influence of different topics and emotions, but it can also analyze the differences in the influence of different user types on the dissemination of topics and emotions. Therefore, we could further study the diffusion mechanism of topics and emotions in social networks when public emergencies occurred.

**Figure 6:**
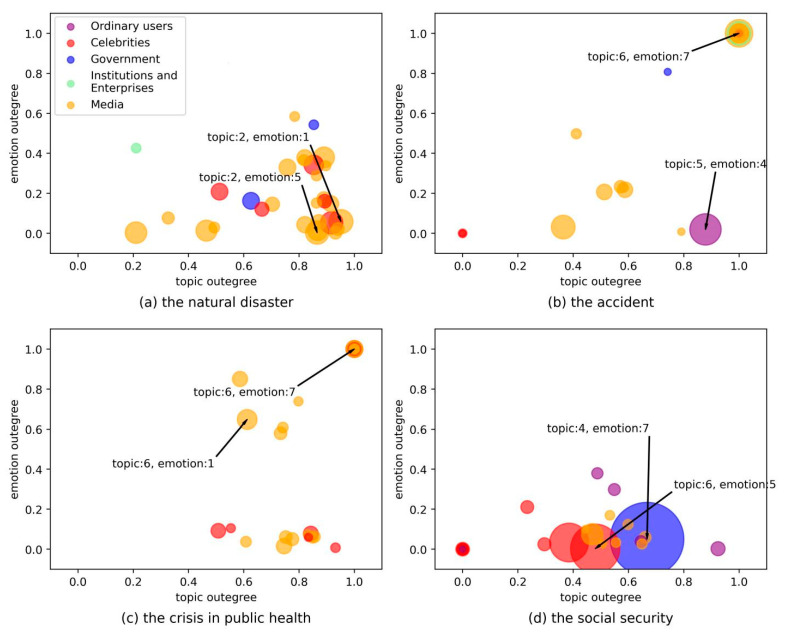
The topic out-degree and emotion out-degree of main user types.

Figure 6a–d shows the differences in topical influence and emotion contagion of nodes of different user types in the natural disaster, accident, public health event, and social security event. By comparing the four figures, we found that celebrities and media users had the largest number of core nodes in social networks. This means that when the topics and emotions related to public emergencies were spread in social networks, celebrities, and media users took an absolute advantage in topical influence and emotion contagion. This was also in line with the research expectation that opinion leaders had the right to speak and had a high influence on social networks. It can also be found in the figure that when the accident occurred, some influential ordinary users were in a dominant position in the propagation of the topic. When the social security event occurred, the government and some ordinary users played an important role in the topic propagation and emotion contagion. We found that the impact of topic and emotion was influenced by the types of users, which means users′ authority, social prestige, and other factors had an impact on the topic propagation and emotion contagion.

Through the calculation of the topic out-degree, we also found that when various types of public emergencies occurred, the influence of the topic types of core nodes in social networks was different. In the natural disaster event, topic 2 (mourning and honoring the hero of firefighting) attracted the most attention, and the tribute to the firefighting hero became the focus of transmission in social networks. When the accident occurred, the topic types that attracted the most attention in social networks were topic 5 (pray for the safety of the affected people) and topic 6 (discussion on the cost of quarantine), respectively, which contained the public′s blessing and concern for the trapped people. When the public health event occurred, network users were most eager to discuss topic 6 (confirmed situation of cruise ship pneumonia) related to the event, and the understanding of the specific situation of the emergency became the focus of the spread of the topic in social networks. When the social security event occurred, topic 4 (police officers knelt on the ground) and topic 6 (relatives of hostages held in Kunming deny rumors) attracted the public′s attention most and aroused wide discussion.

Figure 6 also shows the difference in the emotional influence of core nodes when different types of emergencies occur, based on the calculation of emotion out-degree. We found that when the natural disaster occurred, the most influential core nodes in social networks show the public emotions of like and fear, respectively, which were expressed as the love for the fire heroes and the fear of the disaster. When the accident occurred, the public emotions were happy and angry in the core nodes. Happiness was expressed as the recognition of the handling method and rescue speed, the anger for the collapse caused by the illegal reconstruction of the hotel house. When the public health event occurred, the public shows the emotions of anger and like in the core nodes. Anger represented the behavior of the ship’s lack of responsibility for the spread of the epidemic, while like was the recognition and support of the country′s strict control of the epidemic. When the social security event occurred, fear and anger were expressed in the core nodes. Fear was expressed as the concern for the life safety of the abducted students, and anger came from the perpetrator′s wanton harm to others.

By calculating the topic out-degree and emotion out-degree, we further explored the diffusion mechanism of topic and emotion in social networks. It was found that, although the types of public emergencies selected were different, there was something in common between topics and emotions in the propagation of social networks when emergencies occurred. In the topic propagation, we found that the topic types that the public paid the most attention to in social networks focused on the understanding of the event situation and the rescuer and the victim when the event happened. In the emotion contagion, we found that when public emergencies occurred, the public′s overall emotion types in social networks were mainly negative emotions. However, positive emotions would also be generated due to the excellent qualities of the event-related objects in public emergencies. The bubbles in each figure are mainly located at the lower right of the figure, which indicates that the out-degree of topic was often greater than the out-degree of emotion. The topic information was more easily transmitted than the emotion in public emergencies.

### 5.5. Coevolution of Topic and Emotion

In this paper, we constructed the diffusion maps of related topics and emotions in social networks when different types of public emergencies occurred using topic recognition and emotion analysis. Through the calculation of topic out-degree and emotion out-degree, the topical influence and emotion contagion mechanism of the core nodes in social networks constructed by forwarding relationships were explored when an emergency occurred. It was of great significance for us to understand the propagation mechanism of public emergencies in social networks, maintained network security, and realized emergency management. At the end of this study, the paired sample t-test was used to test the significance of the mean differences of topic out-degree and emotion out-degree in different types of emergencies based on excluding isolated nodes. In addition, we used the method of correlation test to further explore whether the topic and emotion of different user types were related to each other in the evolution trend of social networks when public emergencies occurred.

Firstly, the paired sample t-test was used to test whether there was a significant difference in the mean value of the topic exposure and emotion exposure when different types of public emergencies occurred. When there was a significant difference between the mean values, it proved that topic and emotion did not converge in the process of propagation and had their propagation characteristics. When there was no significant difference, topic and emotion had a more consistent evolution mechanism in the process of propagation. The test results are shown in Table 6. We found that when different types of public emergencies occurred, the mean values of topic out-degree and emotion out-degree were significantly different. Compared with emotion types, the propagation of topic categories in social networks was more similar.

Secondly, we tested the correlation between topic out-degree and emotion out-degree when different types of emergencies occurred, to explore the relationship between topic and emotion in the propagation process of social networks. The test results are shown in Table 7. We found that when different types of public emergencies occurred, although topic and emotion had different mechanisms in the propagation process, there was still a positive correlation between them at the statistical level. A high level of topic influence was often accompanied by a high level of emotion appeal, and the spread of topic and emotion in social networks had the possibility of co-evolution.

Finally, we divided users in social media into different types and compared the correlation degree of different types of users in terms of topic and emotion and explored the differences of topic diffusion and emotion contagion among different types of users when different types of public emergencies occurred. The test results are shown in Table 8. The research found that for the five types of social network users, the topic out-degree and emotion out-degree were positively correlated. Social network users with a high level of topic influence were more likely to have a higher level of emotional appeal, which further confirmed the co-evolution of the topic and emotion in social networks. In addition, by comparing the correlation levels of various types of users, it can be seen that the correlation coefficients of ordinary users were all greater than the overall correlation level, and the correlation coefficient of media was relatively low. This was because ordinary users’ social influence was relatively small, content forwarding times were few and limited within the social circle, the forwarding content mainly revolved around the original microblog. While the media had a large fan base and great social influence. In the social media platform of square communication, the topic and emotion forwarded by the media were more likely to deviate.

## 6. Conclusions

We took different types of public emergencies as research objects and used social networks as a social sensor when public emergencies occurred. In addition, topic recognition and emotion analysis were adopted to explore the propagation mechanism of event-related topics and emotions in social networks when public emergencies occurred. Finally, visualization software was used to construct the propagation maps of the topic and emotion. To realize the recognition and extraction of relevant topics contained in text data, we first searched event keywords in social media in a limited period to obtain relevant data information and preprocessed the text data. Second, the text data were transformed into word vectors by the Skip-gram model. Finally, the K-means clustering method was used to realize the recognition of topic features and obtain topic feature words. To classify the emotions contained in textual data, we used a variant of the bidirectional long short-term memory (biLSTM) network to classify emotions into seven types based on training datasets, such as anger, and sadness.

For research question 1, on the one hand, this study found the commonality among different emergencies in the topic and emotion communication. For the topic, rescue, accountability, and mourning were common topics in emergencies. For emotions, negative emotions, such as sadness and nausea, were more likely to spread in emergencies. On the other hand, this study found diffusive differences in topic and emotion of various types of emergencies. Natural disaster events involve the relationship between human beings and nature, and the conflict involved in the events was relatively single, which was easy to trigger the public to pay tribute to the rescuers. Public health events were prone to panic and heartbreak due to their potential harmfulness, while they were prone to anger over the topic of inadequate prevention and management. Since accidents and disasters reflect social contradictions and other real social problems, their discussions focused on the central node, which was more likely to lead to group polarization. Discussion of social security incidents was fragmented, as the media and the government may have played down the issue in order to reduce panic.

For research question 2, this study found that the topic and emotion propagated simultaneously. Topic information diffused more easily than emotion. The collaborative diffusion of topic and emotion among releasing subjects was different. The topic–emotion collaborative diffusion relationship of media’s topic and emotion was relatively poor. The topic-emotion collaborative diffusion relationship of ordinary users and celebrities was more synchronized. However, this study also found that the media, government had advantages in the scale of information diffusion. Thus, it can be inferred that the multi-level diffusion relationship of the public emergencies. The information of the emergency event receives wide attention through the amplifier function of media and government social media accounts, and then celebrities and ordinary users as participants conduct close topic and emotion communication.

The findings of this study have some practical significance for emergency management. Firstly, according to the topic and emotion characteristics of emergencies, the emergency response department can formulate emergency plan according to the public opinion topic and emotion type that may appear in various types of emergencies. For example, coverage and recognition of the heroism of aid workers can be used to reduce public discontent and stabilize social order. Then, relevant departments should pay attention to the differences in the emotional diffusion ability of different nodes. When dealing with public opinions about events, emergency management departments should take differentiated measures, pay attention to the topical content and factual information revealed by the media and information released by departments, and guard against the abnormal guidance of celebrities’ opinions on public attention and emotion.

As an exploratory study, this study has some limitations. First of all, this study only selected a single event case as a representative under various categories. The conclusions drawn by this study need to be verified on a larger event case set. Secondly, the field of natural language processing is developing rapidly, and the accuracy level of the topic clustering and emotion mining methods used in this study still needs to be further improved. Therefore, further research can expand the case event data and integrate time, geographical location, and other social media information to explore the co-evolution mechanism of the topic-emotion.

## Figures and Tables

**Table 1:** The numbers of microblog entries and forwarding relationship at each event.

Event Type	Microblog Count	Forwarding Relationship Count	Extraction Date	Searched Hashtag
N ^1^	103,804	99,413	30 March 2019–10 April 2019	#forest fire in muli county, sichuan #, #forest fire in liangshan#, #bodies of 30 missing fire fighters in sichuan found #, # The forest fire in muli, sichuan has been put out#
A	101,369	95,608	7 March 2020–20 March 2020	#A hotel collapses in Quanzhou, Fujian province#, #A list of the victims of the collapsed hotel in Quanzhou#, #Quanzhou hotel collapse side elimination kill side rescue#, #Quanzhou hotel collapse rescue#
C	99,250	93,715	19 January 2020–21 February 2020	#Diamond Princess#, #Diamond Princess has 40 Americans with confirmed infections#, # Diamond Princess Added 41 COVID-19#, #Diamond Princess 14 Chinese infected with COVID-19#
S	127,444	124,824	22 January 2021–10 February 2021	#Hostage-taking incident in Kunming #, #Rescued student in Kunming receiving treatment #, #Kunming female reporter came to talk to calm the suspect#, #One dead, seven injured in Kunming hostage-taking#

^1^ N denotes the natural disaster, A denotes the accident, C denotes the crisis in public health, and S denotes the social security.

**Table 2:** Event types and the criteria for node filtering.

Event Types	Criteria for Node Filtering
N	degree ≥ 4
A	degree ≥ 6
C	degree ≥ 8
S	degree ≥ 6

**Table 3:** The numbers of user types at each event.

User Types	Number of Nodes of Users			
	N	A	C	S
Ordinary users	68	98	71	264
Celebrities	228	218	309	405
Government	284	163	20	21
Institutions and Enterprises	26	7	28	6
Media	369	232	344	45
Total	975	808	772	741

**Table 4:** The main attributes of the forwarding networks at each event.

Attributes	N	A	C	S
Average degree	0.094	0.141	0.114	0.574
Network diameter	3	3	3	8
Modularity	0.935	0.947	0.977	0.928
Weakly Connected components	888	696	685	320
Strongly Connected components	973	808	772	740
Average eigenvector Centrality	0.012	0.019	0.015	0.228
Average path length	1.083	1.141	1.158	2.153
Number of nodes	975	808	772	741
Number of edges	92	114	88	425

**Table 5:** Topics and the feature words of high frequency.

Category	No.	Topic Summary	Feature Words of High Frequency (Partial)
N	1	Condemning the net users who insult firefighters	Kindling, compatriots, consequences, platform, diary, loss, doubt, chilling, heroic, helpless, figure, struggle
	2	Mourning and honoring the hero of fire fighting	Sacrifice, farewell, forest, Sichuan, backfire, hero, mourning, mourning, salute
	3	Sorrow for the dead firemen	Recognition, red seal, get through, process, negative, sorrow, vitality, tell, rescue
	4	The surviving firemen developed stress reactions	Receive, open, source, fire brigade, hometown, relatives, assistance, compensation
	5	The body of the missing firemen was recovered.	Inside, search and rescue, altitude, fire fighting, loss, misfortune, fireman, wind, death
A	1	Shocked that the collapse had occurred	Companion, essence, commerce, morality, order, fault, healer, pit
	2	The rescue picture express the emotion and life	The answer, team leader, survival, buddy, emergency, urine, casualty, observation point, rescue, survivors
	3	In other events, a doctor from Guangdong died in a car accident	Backup, pure water, burden, universal, screenshot, prevention, full load, adhere to the principle, communication
	4	Search and rescue situation of Quanzhou Hotel	Building, hospital, express hotel, tension, death, physical signs, treatment, trapped, ruins
	5	Pray for the safety of the affected people	Candle, rest, price, sadness, sadness, guard, expectation, heartbreak
	6	Discussion on the cost of quarantine	Demonstration, typical, income, antipyretic, illness, weakness, depression, remediation
	7	Quanzhou hotel collapse investigation	Endure, infrastructure, villains, intervention, help, position, vision, hurt, blessing
C	1	Netizens condemned Japan’s lax control of the epidemic	Soaring, lost, argument, male doctor, detained, health bureau, military, medical
	2	Speculation over what happened to the Diamond Princess	Ensures, Visits, Hard-hit Areas, Calculations, Living Hell, Response, Rescue Team, Identity
	3	Sneering at the institutional shortcomings of Western countries	Guards, no move, rights and interests, ridicule, thriller, magic mirror, abuse, flout, all walks of life
	4	Domestic and foreign government epidemic prevention and control information	Reported number, cough, medical treatment, aggravation, program, media reports, territory, confirmed, suspected
	5	Condemning the inaction of relevant government departments	The failed investigation, leave, indefinite, mental health, traceability, death penalty, elimination, peak
	6	The confirmed situation of cruise ship pneumonia	Latest news, virus detection, virus infection, release, news, total, press conference, pneumonia
S	1	Condemn the prisoner, sympathize with the child	information, fear, images, cover-ups, harm, villains, hatred, criminals
	2	Netizens praised the children for their bravery	Sigh, end, schoolboy, foil, influence, fame
	3	A female reporter approached the hostage-taker to calm him down	Witnesses, key moments, media coverage, tributes, SWAT, emotions, mitigation
	4	Police officers knelt on the ground	Captured, swapped, taken hostage, hero, calm down
	5	Internet users supported the police and the female journalists	Motivation, relatives, law, manners, determination, guard, face, responsibility
	6	Relatives of hostages held in Kunming deny rumors	Press conference, unwritten rules, bravery, answer paper, little boy, hostage, gangster
	7	Family members spoke out in response to the details of the hijacking	Criminal behavior, bullying, Kunming city, student, shooting, criminal behavior, suspect, family member

**Table 6:** The paired sample T-test of topic out-degree and emotion out-degree.

Types	Mean		T Value
	Topic Out-Degree	Emotion Out-Degree	
N	0.552	0.240	55.344 **
A	0.430	0.342	26.678 **
C	0.395	0.276	30.955 **
S	0.043	0.019	16.769 **

Note: ** *p* < 0.01.

**Table 7:** The interrelation test of topic out-degree and emotion out-degree.

User Types	The Different Types of Public Emergencies			
	N	A	C	S
Total	0.706 **	0.887 **	0.833 **	0.815 **

Note: ** *p* < 0.01.

**Table 8:** The interrelation test of different user types on topic out-degree and emotion out-degree.

User Types	The Different Types of Public Emergencies			
	N	A	C	S
Ordinary users	0.853 **	0.970 **	0.915 **	0.818 **
Celebrities	0.753 **	0.905 **	0.841 **	0.801 **
Government	0.627 **	0.769 **	0.741 **	0.662 **
Institutions and Enterprises	0.759 **	0.856 **	0.731 **	0.946 **
Media	0.212 **	0.634 **	0.439 **	0.419 **

Note: ** *p* < 0.01.

## Data Availability

Data sharing not applicable.
